# Spontaneous Regression of Metastatic Lesions of Adenocarcinoma of the Gastro-Esophageal Junction

**DOI:** 10.7759/cureus.18784

**Published:** 2021-10-14

**Authors:** Richard Mitchell, Amandeep Kaur, Foma Munoh Kenne, Ahmed Khan, Wahib Zafar

**Affiliations:** 1 Internal Medicine, Richmond University Medical Center, Staten Island, USA; 2 Hematology and Medical Oncology, Richmond University Medical Center, Staten Island, USA; 3 Internal Medicine, Brookdale University Hospital Medical Center, Brooklyn, USA; 4 Radiology, Richmond University Medical Center, Staten Island, USA; 5 Hematoloy and Oncology, Richmond University Medical Center, New York, USA

**Keywords:** adenocarcinoma of esophagus (ac), pet-ct with 18f-fdg, immune repose, regression, spontaneous

## Abstract

Spontaneous regression of cancer is a rarely recognized entity in modern medicine. Historically, this was recognized and hypothesized that an infection causes immune activation, indirectly stimulating the body to destroy tumor cells. Similarly, immune-oncology has now become a major modality in the treatment of solid and some liquid malignancies. However, now with improved therapeutic modalities in the oncology world, one does not get to appreciate our own immune system’s ability to fight cancer. We present a patient who had spontaneous regression of metastatic adenocarcinoma of the gastroesophageal junction (GEJ). The patient is a 58-year-old female who had presented with early satiety and dysphagia for which she underwent esophagogastroduodenoscopy which showed an esophageal mass and endoscopic ultrasounds (EUSs) confirmed adenocarcinoma of the GEJ with metastasis to the regional lymph nodes and left supraclavicular lymph nodes. The patient had refused to undergo any surgical, medical oncological, or holistic treatments. Interim disease monitoring positron emission tomography-computed tomography (PET-CT) showed resolution of the metastatic sites of gastroesophageal cancer with clinical improvement of her symptoms. She continues to have this distant regression of metastatic gastroesophageal cancer six months after the initial diagnosis. In literature, spontaneous cancer regression has been reported in melanoma, renal cell carcinoma, and basal cell carcinoma. To our knowledge, this is the first case reported of spontaneous regression of metastatic lesions involving adenocarcinoma of the GEJ with no medical or surgical intervention.

## Introduction

Spontaneous regression of cancer is defined as the partial or complete disappearance of primary tumor tissue or its metastases in a patient who never received cancer-directed treatment. There has been a significant increase in the available treatments for cancer. One of them is the development of immunotherapy which essentially uses the ability to fight off cancerous cells which tends to otherwise escape the immune-directed killing [1-2]. The immune phenomenon is presumed to be one of the explanations for the spontaneous regression of cancer. It has been postulated that the stimulation of the immune system leads to the destruction of cancer cells, in turn causing tumor regression [3]. Spontaneous regression of cancer is an uncommon phenomenon but has been observed for hundreds of years [4]. Spontaneous regression of esophageal cancer is even more rare, with only a few cases having been reported in the literature [5-6]. We present a 58-year-old female with poorly differentiated adenocarcinoma of the gastroesophageal junction (GEJ) with multiple lymph node metastases, who had resolution of the lymph node metastases without regression of the primary lesion four months later without any cancer-directed therapy. This phenomenon is characterized as clinical category two of the Everson categorization of the spontaneous regression of the pathologically proven distant metastasis. She continues to have this distant regression on positron emission tomography-computed tomography (PET-CT) seven months after the initial diagnosis, with continued clinical stability nine months after her initial diagnosis. The patient is encouraged to pursue oncological treatment on every visit, however, she continues to refuse any oncological intervention for the primary cancerous lesion. 

## Case presentation

Our patient was a 58-year-old female who initially presented in November 2020 with a history of dysphagia and early satiety. Her past medical history was significant for gastric bypass done in 2001, multiple ventral hernia repairs, B12 deficiency, and depression. She underwent esophagogastroduodenoscopy (EGD) and was found to have a one-centimeter polyp in the distal esophagus. Biopsy of this polyp revealed a poorly differentiated adenocarcinoma of the GEJ, no evidence of deficient mismatch repair (low probability of MSI-H) and it was HER-2 positive. Initial PET-CT showed 1.6 cm fluorodeoxyglucose (FDG) avid lateral wall thickening of the distal end of the esophagus with SUV 3.1, with another focus at the GEJ SUV 2.4, also noted were 2 cm x 1.6 cm left supraclavicular lymph node SUV 7.2, 1 cm precarinal lymph nodes, and other nodes included retrocrural, gastrohepatic, periaortic, and aortocaval lymph nodes. EUS showed a medium-sized ulcerating mass measuring two centimeters at GEJ extending to the gastric pouch. There was also invasion of the muscularis propria. Five malignant-appearing lymph nodes were visualized in the lower para esophageal mediastinum, celiac, and peri-aortic regions.

**Figure 1:**
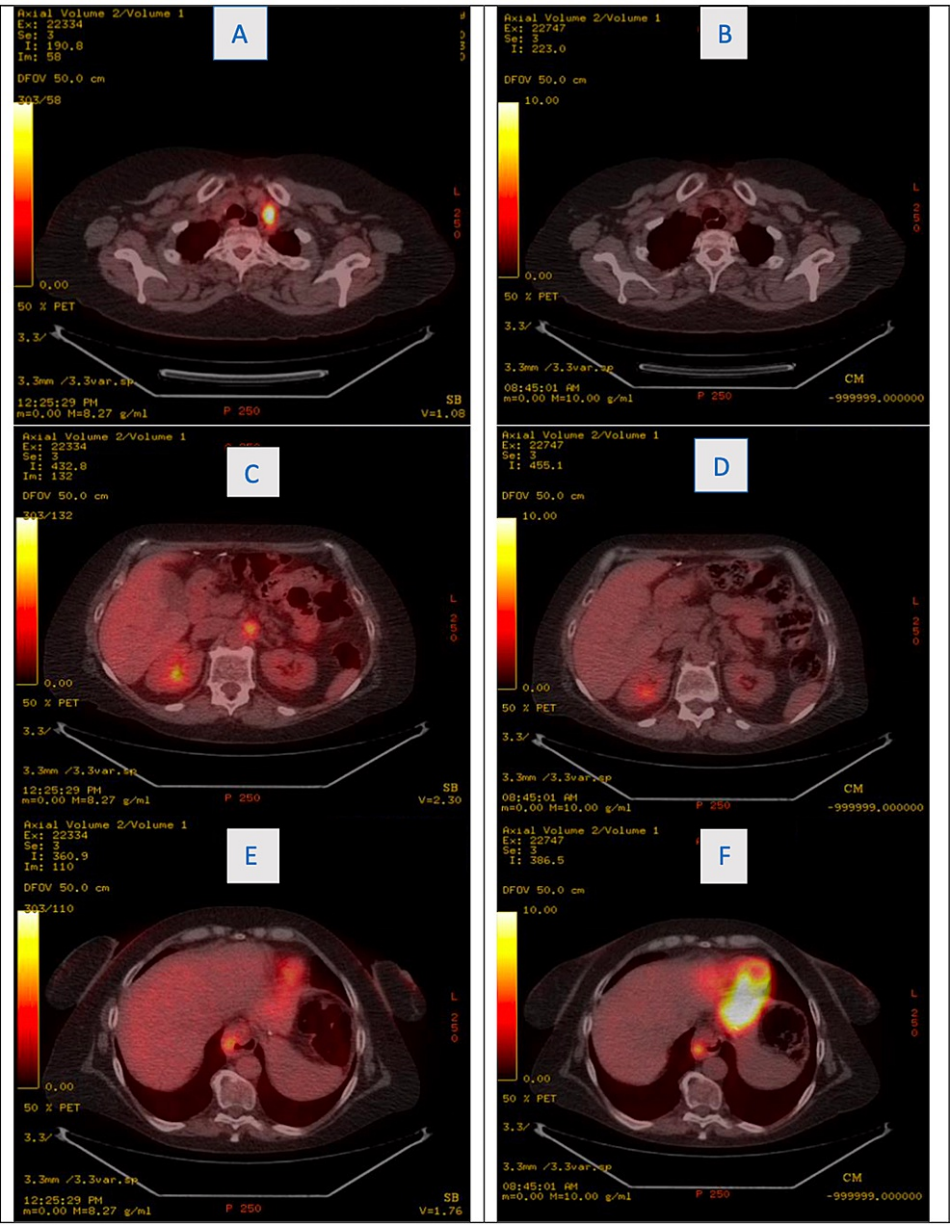
A and B: Interval resolution of supraclavicular lymph node in the neck between December 26, 2020 (A) and April 26, 2021 (B). C and D: Interval resolution of additional para-aortic lymph node in the abdomen between December 26, 2020 (C) and April 26, 2021 (D). E and F: Progression of esophageal mass with interval increase in SUV between December 26, 2020 (E) and April 26, 2021 (F). SUV, standardized uptake value

Fine needle aspiration of the para-aortic lymph node was positive for metastatic adenocarcinoma. Given these findings, the patient was staged as T3N2M1a. Her combined positive score (CPS) score was 20, and she was offered palliative chemotherapy and immunotherapy. The patient expressed wishes not to pursue any cancer-directed therapies and had no changes in her diet or had other holistic measures. A PET-CT was repeated four months later which showed findings consistent with known primary malignancy but anatomic and metabolic resolution of metastasis to the left supraclavicular lymph node, the intraabdominal lymph nodes, and hypermetabolic focus of GEJ that was previously seen (Figure 1). The size of the supraclavicular lymph node and the other gastric and para-aortic lymph nodes significantly decreased in size and were no longer metabolically active although, the primary lesion had increased in size. The patient had another PET-CT almost seven months later from her initial diagnosis, which showed findings very similar to the PET-CT shown below, performed about five months from her initial diagnosis. She did not receive any treatments or medical care at any time between diagnosis and the repeat PET-CT. Nine months after her initial presentation, the patient reports symptomatic resolution of dysphagia. The patient is encouraged to obtain oncological treatment for her persistent primary cancerous lesion at the GEJ; however, she refuses any medical or surgical treatment options at this time.

## Discussion

Esophageal cancer has two main subtypes: squamous-cell carcinoma and adenocarcinoma. While squamous-cell carcinoma makes up the majority of esophageal cancer worldwide, adenocarcinoma has become the predominant subtype in the western world. The incidence of esophageal adenocarcinoma in 1975 was 0.4 per 100,000 compared to 2009, which was 2.6 per 100,000 [7]. The spike in cases has been attributed to the increase in obesity and gastroesophageal reflux disease. A high BMI index increases the risk of esophageal adenocarcinoma by a factor of 2.2 [8]. Adenocarcinoma is more prevalent in males than in females. 

For esophageal carcinoma, it is crucial to determine the extent of lymphatic involvement. Our patient had extensive lymph node involvement with a PET-CT showing six regions involved and EUS confirming five malignant-appearing nodes. The presence and extent of lymph node involvement have a prognostic value -- greater than four having a poorer prognosis [9]. Two lymphatic plexuses are present in the esophagus, and lymphatic fluid may move up, down, or bidirectional. Thus, fluid may move to any nodal bed and region of the thorax. Studies have shown esophageal carcinoma metastasize to cervical, thoracic, and abdominal lymph node stations, regardless of the primary tumor location [10]. One particular study showed in GEJ tumors, abdominal lymph nodes were positive in all, thoracic lymph nodes were positive in 40%, and cervical lymph nodes were positive in 20% [11]. A possible explanation for the involvement of cervical lymph nodes in GEJ tumors could be the presence of an extensive lymphatic network in the submucosa and even in the lamina propria of the esophagus, with both intramural and longitudinal lymphatic drainage [10]. Another interesting phenomenon is seen in esophageal carcinoma -- skip metastasis. Skip metastasis are distant lymph nodes that bypass the first lymph node and directly metastasize into the second or third lymph node. Patients with skip metastasis had a significantly better five-year survival rate than patients with continuous metastasis [12]. 

Spontaneous regression of cancer is defined as the partial or complete disappearance of primary tumor tissue or its metastases in a patient who never received cancer-directed treatment. There are four clinical categories as defined by Everson: 1) regression in the primary tumor, 2) regression of metastatic tumor (confirmed via histology), 3) regression of metastatic tumor (no pathological confirmation), and 4) regression of presumptive metastases by radiography [13]. Our patient could be classified as category two since she had fine needle aspiration (FNA) positive for adenocarcinoma. Spontaneous tumor regression is an uncommon phenomenon but one that has been observed for hundreds of years. It was initially known as St. Peregrine tumor, being named after Peregrine Laziosi, who developed a bone tumor of his tibia that spontaneously disappeared. In 1966 Everson and Cole wrote about 176 cases of spontaneous regression from 1900 to 1964. Cole went on to publish additional works in the Journal of Surgical Oncology to address spontaneous regression. He speculated that there could be antigens in our body that stimulate our immune system, causing regression of cancer [13]. Factors associated with spontaneous regression primarily include apoptosis, immune system, and conditions in the tumor microenvironment, particularly the presence of inhibitors of metalloproteinases and angiogenic factors and decreased epithelial cadherin proteins [14]. Common infections have also been implicated in the role of spontaneous regression. Coley, in 1891, wrote about the inoculation of his patients with erysipelas and its curative effect [15]. Since this, many other studies have been done to find the underlying mechanism. It has been postulated that the stimulation of the immune system activates resting dendritic cells, lymphocytes, and natural killer cells, increasing the immunorecognition of tumor cells and leading to the destruction of cancer cells, in turn causing tumor regression [16].

Spontaneous tumor regression is much less reported in esophageal cancer. Cancers most likely to undergo spontaneous tumor regression include melanoma and cutaneous basal cell carcinoma, testicular germ cell, and renal cell carcinoma. Of the reported cases, the subtypes include squamous cell carcinoma and small cell carcinoma [5-6, 17]. Our case is unique in that spontaneous regression occurred in our patient who has esophageal adenocarcinoma. To our knowledge, this is the first case of esophageal adenocarcinoma, which had spontaneous regression in its metastatic lymph nodes without any surgical resection of the primary. 

## Conclusions

Spontaneous regression of cancer is an uncommon phenomenon where either the primary or the metastatic lesions disappear without oncological intervention. We attribute this phenomenon to immune-directed cancer cell destruction. We presented a 58-year-old female who had biopsy-proven poorly differentiated adenocarcinoma of the GEJ with multiple lymph node metastases and had resolution of metastatic sites without any oncological treatment. We encourage this entity to be recognized. However, we do not recommend spontaneous waiting and watching as an alternative to definitive therapeutic modalities. However, if a patient does not wish for treatment at the time of diagnosis, we recommend doing surveillance without any immunosuppressive therapies. If there happens to be a regression of the tumor, then a different treatment strategy could be proposed to the patient, and the patient should be given the choice of all available modalities.
